# GSK3 inhibitor suppresses cell growth and metabolic process in FLT3-ITD leukemia cells

**DOI:** 10.1007/s12032-022-01899-2

**Published:** 2022-12-08

**Authors:** Jing Xia, Shuxian Feng, Jian Zhou, Lin Zhang, Dingfang Shi, Mengjie Wang, Yi Zhu, Chaozhi Bu, Daming Xu, Tianyu Li

**Affiliations:** 1 Department of Pediatric Laboratory, The Affiliated Wuxi Children’s Hospital of Nanjing Medical University, Wuxi, 214000 Jiangsu China; 2 Department of Hematology & Oncology, The Affiliated Wuxi Children’s Hospital of Nanjing Medical University, Wuxi, 214000 Jiangsu China; 3 Center of Reproductive Medicine, State Key Laboratory of Reproductive Medicine, Research Institute for Reproductive Health and Genetic Diseases, The Affiliated Wuxi Matemity and Child Health Care Hospital of Nanjing Medical University, Wuxi, 214002 Jiangsu China

**Keywords:** GSK3, AML, FLT3, BIO, Metabolism

## Abstract

Glycogen Synthase Kinase-3 (GSK-3) was recently implicated in the dysregulated biology of acute myeloid leukemia (AML). Low concentrations of GSK-3 inhibitors, SB216763 and BIO, suppressed the proliferation of AML cells with FLT3-ITD as early as 24 h after treatment. BIO was used in subsequent assays since it exhibited higher inhibitory effects than SB216763. BIO-induced G1 cell cycle arrest by regulating the expression of cyclin D2 and p21 in MV4-11 cells, and promoted apoptosis by regulating the cleaved-caspase3 signaling pathways. In vivo assays demonstrated that BIO suppressed tumor growth, while metabolomics assay showed that BIO reduced the levels of ATP and pyruvate in MV4-11 cells suggesting that it inhibited glycolysis. BIO markedly suppressed cell growth and induced apoptosis of AML cells with FLT3-ITD by partially inhibiting glycolysis, suggesting that BIO may be a promising therapeutic candidate for AML.

**Supplementary Information:**

The online version contains supplementary material available at 10.1007/s12032-022-01899-2.

## Introduction

Acute myeloid leukemia (AML) is a hematologic malignancy characterized by disordered proliferation and differentiation of progenitor cells in the peripheral blood and bone marrow. Pediatric AML accounts for approximately 25% of all childhood acute leukemia [1, 2]. Despite significant improvement in intensified chemotherapy, risk-adapted treatment and allogeneic stem cell transplantation, AML is associated with high rates of refractory disease, relapse, drug resistance and treatment-related toxicity, which results in poor survival rates and prognosis [3, 4]. Although advanced genomics and molecular biology has increased knowledge on the etiology and pathophysiology of AML and led to improved therapeutic strategies for AML, the precise molecular mechanisms underlying AML development remain unclear.

Mutations of genes such as Fms-like tyrosine kinase 3 (FLT3), DNA methyltransferase 3 alpha (DNMT3A), and ten-eleven translocation (TET) as well as chromosomal abnormalities induced by fusion genes serve as diagnostic and/or prognostic markers, and targets for therapy against AML [5, 6]. Fms-like tyrosine kinase 3 (FLT3) is a class III receptor tyrosine kinase that is aberrantly expressed in 70–100% of AML [7]. FLT3-internal tandem duplications (FLT3-ITD) mutation is the most common type of mutation affecting 25–30% of AML patients [8, 9], including 5–15% pediatric patients [10]. AML cells with FLT3-ITD mutation are characterized by constitutive auto-phosphorylation and factor-independent activation of FLT3 receptors that enhance irregular cell proliferation through various signaling pathways including PI3K/AKT, Ras/MAPK and STAT [11, 12]. Cell lines haboring FLT3-ITD mutations also exhibit a significant reduction in mitochondrial respiration and substantial promotion of glycolysis [13, 14], suggesting that aberrant metabolic alteration could be associated with the pathogenesis of AML.

Glycogen Synthase Kinase-3 (GSK-3) is a serine/threonine kinase involved in glycogen and protein synthesis that is encoded by two different genes: GSK-3alpha and GSK-3beta [15]. GSK3 regulates several biological processes by degrading β-catenin in the ubiquitination pathway through formation of a complex with Axin protein, adenomatosis coli (APC), protein phosphatase 2A and casein kinase 1α. GSK3β inhibitors suppress differentiation and proliferation, and induce apoptosis of AML cells [16, 17]. Intriguingly, leukemia cells are characterized by a relatively distinct metabolism that has led to the therapeutic targeting of glycolysis [18]. Some studies demonstrated that GSK-3β regulates basal glycolysis, glycolytic capacity and mitochondrial respiration in MIA-PaCa-2 cells [19]. It is well-recognized that elevated glycolytic activity provides energy in the formation of adenosine triphosphate (ATP) and carbon skeletons required for tumor growth. The end products of various metabolism pathways are known as metabolites. Inhibition of FLT3 function or mutation of FLT3 gene prevents the binding of ATP to FLT3 kinase domain, thus promoting leukemia cell proliferation and anti-apoptosis process to additional drug treatment [20]. In addition, upregulation of glycolytic enzyme enolase 2 (ENO2) enhanced GSK-3β phosphorylation, subsequently leading to cell proliferation and glycolysis [21]. These findings indicate that GSK3 may be a potential target in AML therapy.

In this study, we found that GSK3 inhibitors distinctively reduced the cell viability of cells harboring FLT3-ITD mutations, and induced G1-phase cell cycle arrest and apoptosis. Moreover, suppression of GSK3 decreased the levels of glycolysis by down-regulating ENO1, and inhibited tumor growth in nude mice. Findings from this study provide valuable insights into the treatment of AML patients.

## Materials and methods

### Cell culture and chemicals

Human AML cell lines MV4-11 and THP-1 were purchased from the ATCC, while RS4;11 and MOLM-13 cell lines were purchased from Beijing Beina Chuanglian Biotechnology Institute (BNCC) and beyotime Biotechnology, respectively. All cell lines were tested for Mycoplasma contamination using the Mycoplasma Stain Assay Kit (beyotime Biotechnology) and grown in standard T-75 flasks at 37 °C (95% air and 5% CO2). RPMI-1640 (Gibco) supplemented with 10% Fetal Bovine Serum (Biological Industries) and 1% penicillin/streptomycin (Gibco) was used for cell culture. GSK-3 inhibitor IX (6-bromo-3-[3-(hydroxyamino) indol-2-ylidene]-1H-indol-2-one, BIO) (Cayman) and SB216763 (sigma-aldrich) were purchased and dissolved in DMSO.

### Western blot analysis

Cells were harvested and lysed in radio immunoprecipitation assay (RIPA) lysis buffer supplemented with protease and phosphatase inhibitors (beyotime Biotechnology). About 40 μg protein per sample was separated using 10% sodium dodecyl sulfate polyacrylamide gel electrophoresis (SDS-PAGE, Epizyme Biomedical Technology) and transferred to nitrocellulose membranes (PALL). The membranes were incubated with 3% bovine serum albumin for 2 h at room temperature (RT) and then incubated overnight at 4 °C with the following primary antibodies: anti-ENO1 (cell signaling technology, 3810), anti-β-Catenin (Cell Signaling Technology, 8480), anti-caspase3 (cell signaling technology, 14220S), anti-p21 (cell signaling technology, 2947S), anti-cyclin D2 (cell signaling technology, 3741S) and anti-GAPDH (cell signaling technology, 5174). Subsequently, membranes were incubated with horseradish peroxidase–conjugated secondary antibodies (anti-rabbit IgG, Cell Signaling Technology, 7074S) for 1 h at RT. The specific proteins were detected using enhanced chemiluminescence reagent (Millipore), and the band intensity quantified using quantity one software.

### Cell viability assay

MV4-11, SHI-1, THP-1, RS4;11 and MOLM13 cells (5 × 10^4^/ml) were seeded in 96-well culture plates in the presence and absence of GSK3 inhibitor and incubated for 24 h, 48 h, 72 h and 96 h. The viability of treated cells was determined using the Cell Counting Kit-8 assay (Vazyme), where 10 μL CCK8 agent was added per well and the cells incubated for 2 h at 37 °C. A microplate reader (Thermo Scientific) was used to measure the absorbance at 450 nm.

### Analysis of cell cycle and apoptosis using flow cytometry

AML cells (1 × 10^6^ cells) were seeded into six-well plates and then treated with GSK3 inhibitor or vehicle control. The collected cells were fixed in 70% ethanol overnight and washed three times with PBS. The cells were then incubated with staining solution (50 g/ml Propidium Iodide (PI), 0.09% sodium azide, and 500 g/ml RNase A in phosphate-buffered saline) for 30 min. The percentage of cells in the G0/G1, S, and G2/M phases was determined using Flow Cytometry (Becton Dickinson). The rate of cell apoptosis was assessed using Flow Cytometry after staining the cells with Annexin V/Alexa Fluor 647 and PI according to the manufacturer’s instructions. All data were analyzed using FlowJo V10 Software.

### Dot-blot assay

First, each empty well was blocked by adding the blocking buffer and incubating for 30 min at RT. The blocking solution was then removed and diluted cell samples added into each well and incubated for 3 h at RT. The samples were aspirated and the wells washed three times with fresh washing buffer I with a 5 min incubation time at RT for each. Thereafter, the wells were incubated twice with wash buffer II, and then the buffer was discarded. The detection antibody cocktail was pipetted into each well, incubated for 2 h at RT, and then aspirated. HRP-anti-rabbit IgG was added to the wells and incubated for 2 h at RT. Afterwards, the membrane in wells were washed with washing buffer I and washing buffer II as previously described. The membrane was then transferred and printed side up onto a sheet of chromatography paper lying on a flat surface. A detection buffer mixture (500 μL) was gently added onto each membrane and incubated for 2 min. The membrane was transferred to a chemiluminescence imaging system with a CCD camera for dot-blot analysis.

### Targeted metabolomics profiling

The cells were harvested, homogenized in 80% methanol by freezing and whirling, and centrifuged at 10,000 × rpm for 20 min. LC–MS/MS analysis was performed using an LC–ESI–MS/MS system (UPLC, Shim-pack UFLC SHIMADZU CBM A system; MS, QTRAP System). Peak chromatographic, parent ion pair information, secondary spectrum data and retention time were analyzed based on metware database. A standard consisting of 61 metabolites involved 3 energy metabolism pathways was utilized to quantify the retention time and identify metabolites.

### Tumor xenografts

Animal studies were carried out in accordance with the guidelines provided by Nanjing Medical University Animal Care. 1 × 10^6^ MV4-11 cells were subcutaneously injected into 6-week-old BALB/c (nu/nu) nude female mice with weight of 22 ± 3 g, which were randomly assigned into two independent groups including 6 mice in each group. Administration of vehicle (normal saline, once two days) or BIO (80 mg/kg, once two days) started at the 4th day after implantation and continued for the duration of the study. The tumor volume was measured once a week according to the following formula: volume = (longest diameter × shortest diameter^2^)/2.

### Pyruvate and ATP levels

Pyruvate levels were measured using the Pyruvate Assay Kit (Cayman) following manufacturer’s recommendation. Briefly, the cells were washed with PBS and then centrifuged at 10,000×*g* for 5 min. The supernatant was discarded, 0.5 ml of 0.25 M metaphosphoric acid (MPA) added to the cell pellet and placed on the ice for 5 min to deproteinate the sample. The suspension was centrifuged at 10,000×*g* for 5 min, the supernatant removed and 25 μL of potassium carbonate added to neutralize the acid. After centrifugation at 10,000×*g* for 5 min to remove any precipitated salts, supernatant was discarded. The sample was diluted in 1:2 with assay buffer that was subjected to microplate reader analysis at 590 nm. ATP levels were determined using ATP Detection Assay Kit (Beyotime Biotechnology) according to manufacturer’s instructions. Enough standard ATP solution was prepared and added to each well to exhaust the background. Subsequently, 20 μL of sample or standard was added to each well and a luminometer was used to measure the relative light unit (RLU) value. The ATP levels of samples were calculated by referring to the standard curve under the same conditions

### Quantitative reverse-transcription PCR (RT-qPCR)

Total RNA from cell samples was isolated using Trizol® reagent (Invitrogen Life Science). The concentration of the RNA was determined using NanoDrop™ 2000 (Thermo Fisher Scientific). PrimeScript™ RT reagent kit (TaKaRa) was used for cDNA synthesis according to the manufacturer’s instructions. Quantitative PCR was conducted using SYBR Premix ExTaq II (TaKaRa) and the 7500 realtime PCR system (Applied Biosystems). Primers for ENO1: 5′-AAAGCTGGTGCCGTTGAGAA-3′ (forward) and 5′-GGTTGTGGTAAACCTCTGCTC-3′ (reverse); GAPDH: 5′-ATGTATGACAATGGACCCTTCC-3′ (forward) and 5′-TCCCTTGCAGGAGTGTCCATGG-3′ (reverse). Glyceraldehyde 3-phosphate dehydrogenase (GAPDH) was used as an internal reference, while the 2-^ΔΔ^CT method was used to calculate the fold changes.

### Statistical analysis

All data are presented as the mean ± standard deviation (SD). Statistical analyses were performed using SPSS 16.0 software. Independent-samples t-tests and one-way ANOVA were used for statistical analysis. *P* < 0.05 was considered statistically significant.

## Results

### GSK3 inhibitors suppress the proliferation of cells harboring FLT3 mutations

**Fig. 1:**
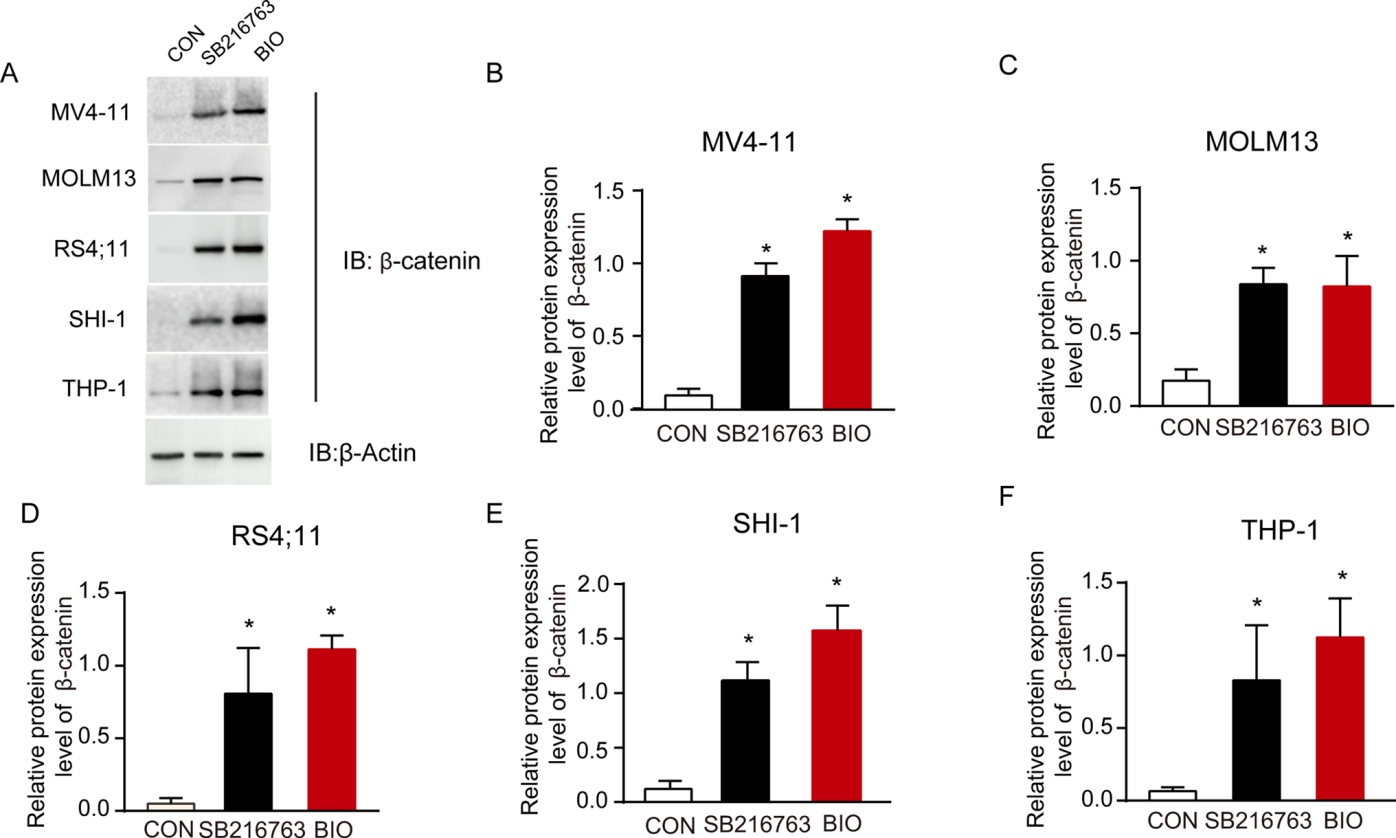
The GSK3 inhibitors up-regulated β-catenin expression in five human AML cell lines cells. **A** Immunoblots analysis showing increased protein level of β-catenin. β-actin was used as a control for sample loading. Quantification of Immunoblot results representing significantly augmented expression of β-catenin following GSK3 inhibitors administration in **B** MV4-11 cell, **C** MOLM13, **D** RS;11 cell, **E** SHI-1 cell, **F** THP-1 cell. *P < 0.05

Since β-catenin is a downstream target of GSK3, we evaluated the inhibitory efficiency of two GSK3 inhibitors by assessing the expression levels of β-catenin using immunoblotting (Fig. 1A). SB216763 (10 μM) and 6-Bromoindirubin-3’-oxime (2 μM) (BIO, GSK3β inhibitor) augmented β-catenin expression in MV4-11 cell, MOLM13, RS4;11 cell, SHI-1 cell and THP-1 cells (Fig. 1B–F) compared to control group, suggesting successful inhibition of GSK3β.

**Fig. 2:**
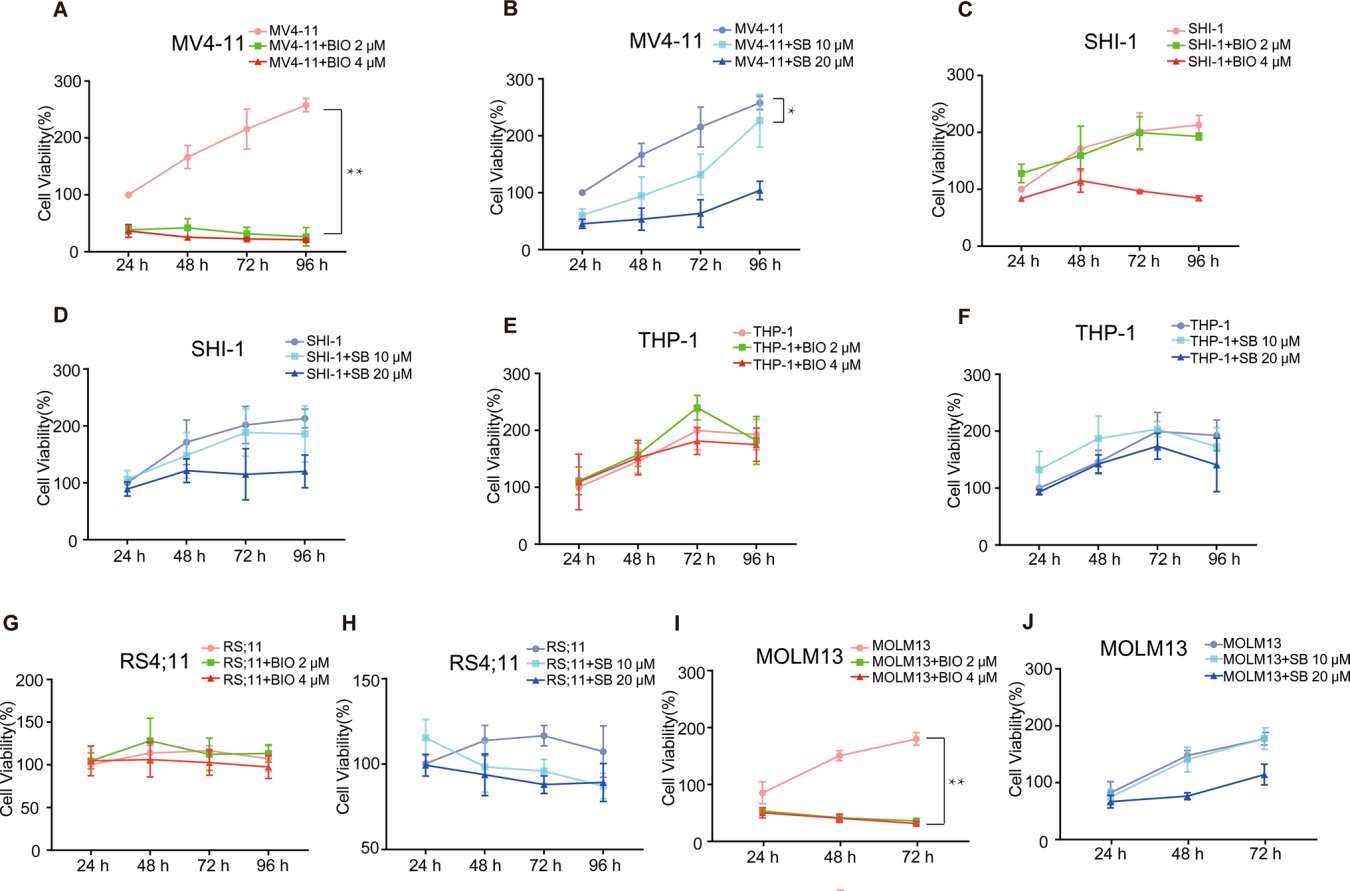
The cell viabilities of five human AML cell lines were suppressed using GSK3 inhibitor IX (BIO) and SB216763. The effects of 2 μM and 4 μM BIO on **A** MV4-11 cell, **C** SHI-1 cell, **E** THP-1 cell, **G** RS;11 cell, **I** MOLM13 cells using CCK8 assaydemonstrated that BIO specially decreased the cell viability of FLT3-ITD mutated cells rather than MLL-rearranged cells. The incubation of 10 μM and 20 μM SB216763 on **B** MV4-11 cell, **D** SHI-1 cell, **F** THP-1 cell, **H** RS;11 cell, **J** MOLM13 cell also particularly showing inhibitory effects on FLT3-ITD mutated cells. *P < 0.05, **P < 0.01 (low concentration of GSK3 inhibitor vs. control)

We then determined the effects of different concentrations of the two independent GSK3 inhibitors, SB216763 and IX (BIO), on the cell viabilities of five AML cell lines. The five AML cells lines were; t(4;11)-positive MV4-11 cell line encoding MLL-AF4 fusion proteins simultaneously with FLT3-ITD mutation, t(4;11)-positive RS4;11 cell line, SHI-1 cell line expressing MLL-AF6 fusion protein, THP-1 cell line with MLL-AF9 rearrangement and MOML13 characterized by FLT3-ITD mutations. We compared the effects of low and high concentrations of GSK3 inhibitors on cell proliferation between MV4-11 and other non-FLT3-ITD mutated cell lines (SHI-1, THP-1 and RS4;11). CCK8 data revealed that low concentrations of the two GSK inhibitors specifically inhibited the proliferation of leukemia cells with FLT3-ITD mutations as early as 24 h after treatment but had no effect on MLL-rearranged AML cells (Fig. 2A–H). BIO exhibited greater inhibitory efficiency than SB216763. Similarly, the two GSK3 inhibitors significantly reduced the proliferation of MOML13 cells (Fig. 2I, J). These results suggested that SB216763 and BIO only suppressed the proliferation of AML cells with FLT3-ITD mutation, and demonstrated that BIO had greater inhibitory effect.

### BIO induces G1 cell cycle arrest and apoptosis in MV4-11 cell

**Fig. 3:**
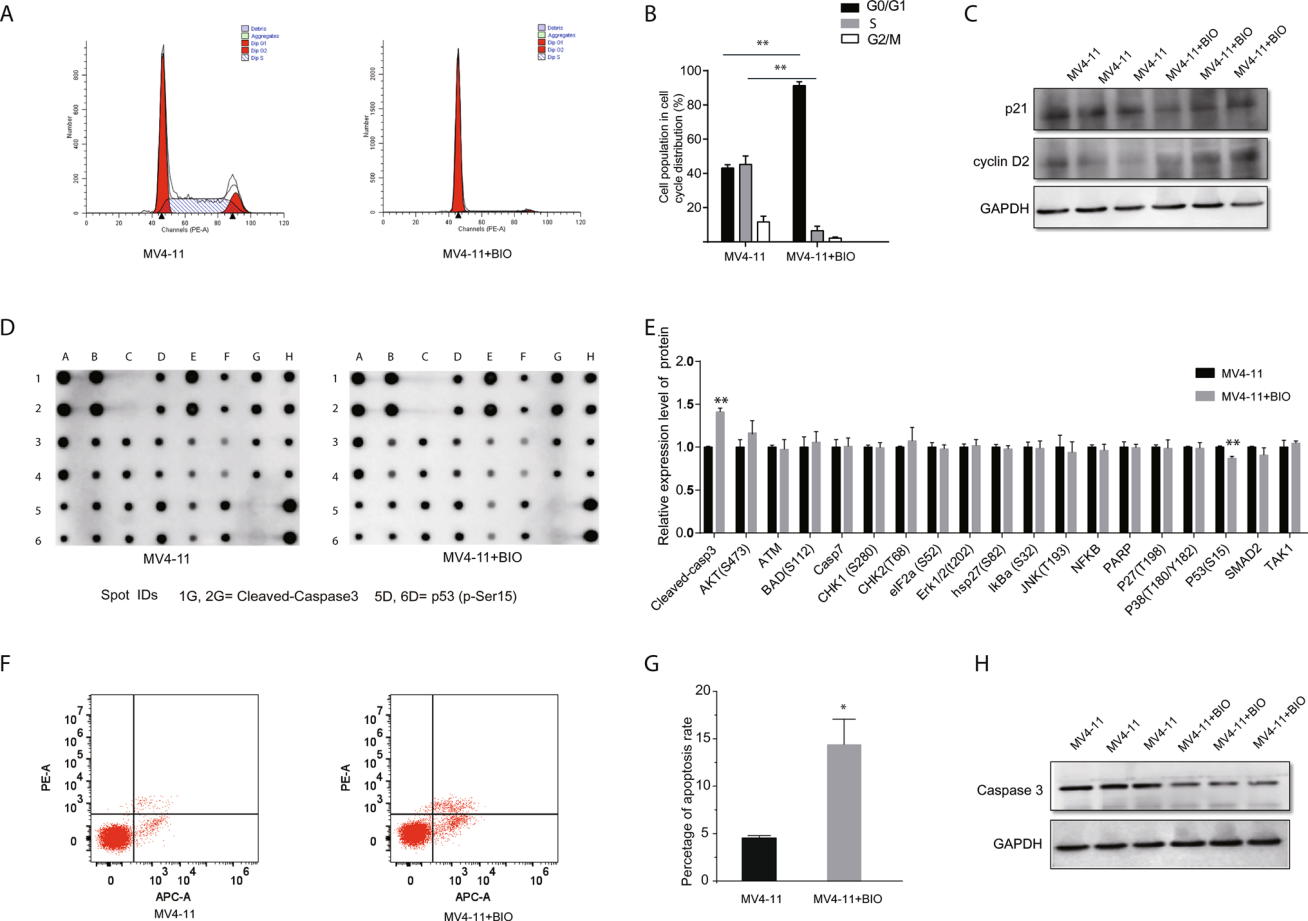
BIO induces G1 cell cycle arrest and apoptosis in MV4-11 cell. **A** The cell cycle progression of MV4-11 cells incubation with and without BIO was measured using PI assay. **B** Quantification of different cell cycle phase in MV4 and MV4 + BIO groups. **C** The protein level related to cell cycle was detected with Western blot. **D**, **E** The expression of 19 intracellular protein related to apoptotic signaling pathways was determined by apoptosis and phosphoprotein antibody array. **F**, **G** Apoptosis of MV4-11 cells following different treatment was detected by flow cytometry after staining with Annexin V-APC and 7AAD. **H** The protein expression of caspase3 were analyzed by Western blot. *P < 0.05

Since FLT3-ITD mutated MV4-11 cells were more sensitive to low concentrations of BIO than SB216763, 2 μM BIO was selected for subsequent analysis. Since BIO significantly suppressed the proliferation of MV4-11, we determined the effect of BIO on cell cycle progression at 24 h using Annexin V/PI staining and flow cytometry. Compared with the control, BIO augmented the proportion of MV4-11 cells in G0/G1-phase, and reduced the proportion of cells in S-phase and G2/M-phase, indicating that BIO-induced G1 cell cycle arrest in FLT3-ITD mutated leukemia cells (Fig. 3A, B). To explore the mechanism by which BIO-induced G1 cell cycle arrest, we investigated the expression levels of cell cycle-related proteins. Western blot results showed that BIO markedly increased the expression of the G1/S-phase marker cyclin D2 and reduced the levels of p21, a major regulator of G1/S-phase transition (Fig. 3C). We also determined the effect of BIO on the apoptotic process of MV4-11 cells, by performing an apoptosis and phosphoprotein antibody dot-blot array to screen the expression of 19 intracellular proteins related to apoptotic signaling pathways. The screening data revealed significant up-regulation of cleaved-caspase3, and down-regulation of p53 (Fig. 3D, E). Moreover, flow cytometry results showed that BIO treatment significantly enhanced the apoptosis of MV4-11 (from 4.5 to 14.5%) (Fig. 3F, G). Results of Western blot assay also indicated that BIO increased the expression of caspase3. Taken together, these data indicated that BIO-induced G1 cell cycle arrest and apoptosis in MV4-11 cell.

### BIO inhibits AML progression in vivo

**Fig. 4:**
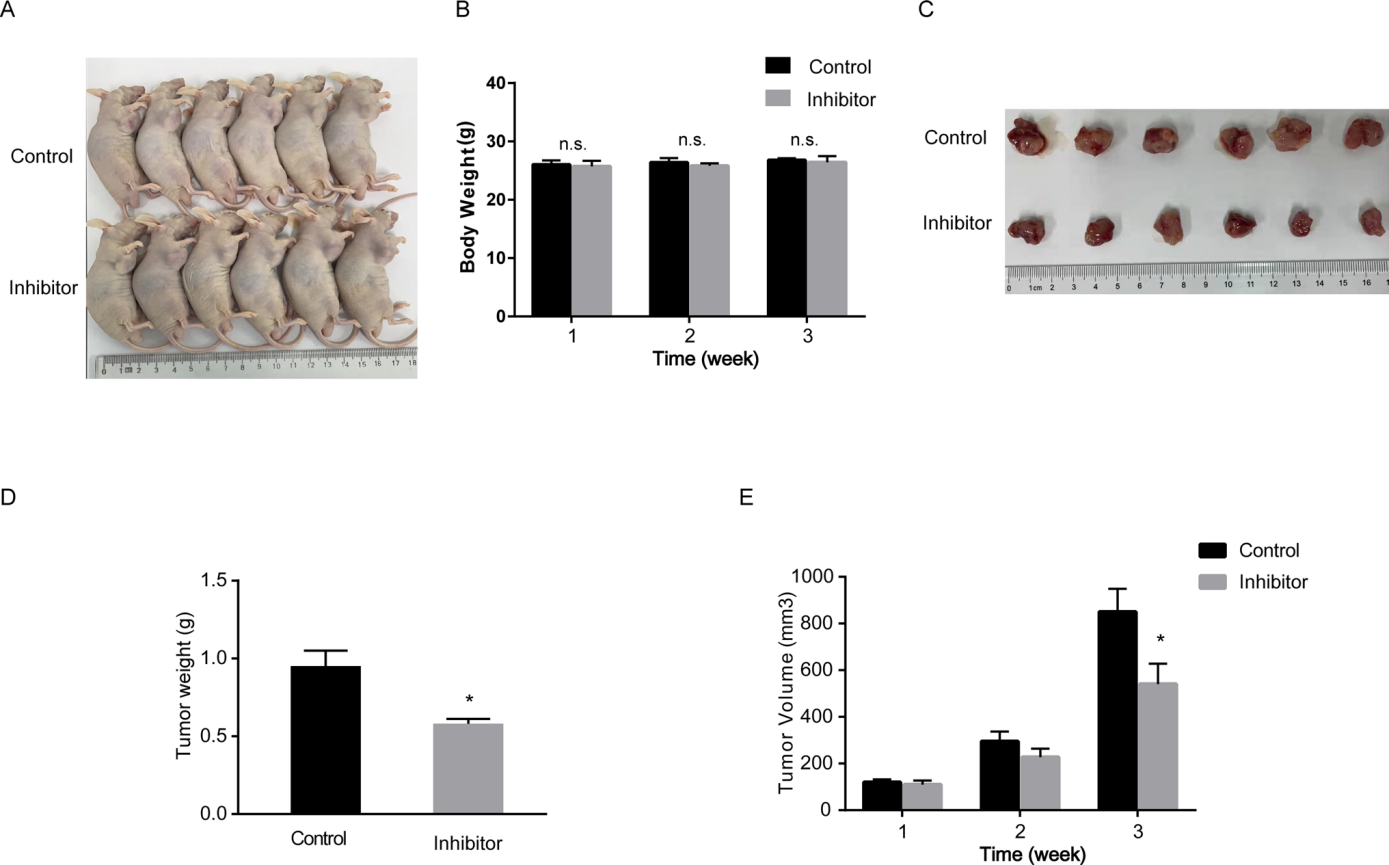
BIO inhibits AML progression in vivo. **A** Representative morphologic images of animals were taken at the day before anatomy. **B** Quantification of bodyweight was analyzed in MV4 alone and BIO-modified MV4 group. The dissected tumor tissues were **C** displayed and **D** measured. **E** The tumor volume in 3 endpoints was determined in control and inhibitor group. *P < 0.05

To further investigate the effect of BIO on AML tumorigenesis in vivo, MV4-11 cells treated with vehicle and BIO were injected subcutaneously into nude mice. The tumor volume was measured every 4 days after injection and tumor tissues were isolated from cell line-derived xenograft mice at study endpoint. Although the body weight of mice in the two groups was not significantly different, (Fig. 4A, B), the tumor volume and tumor weight were significantly lower in the BIO group compared with the control group (Fig. 4C–E). These results indicated that the administration of BIO significantly suppressed tumor growth in vivo.

### BIO suppresses the altered metabolite level involved in glycolysis in MV4-11 cells

**Fig. 5:**
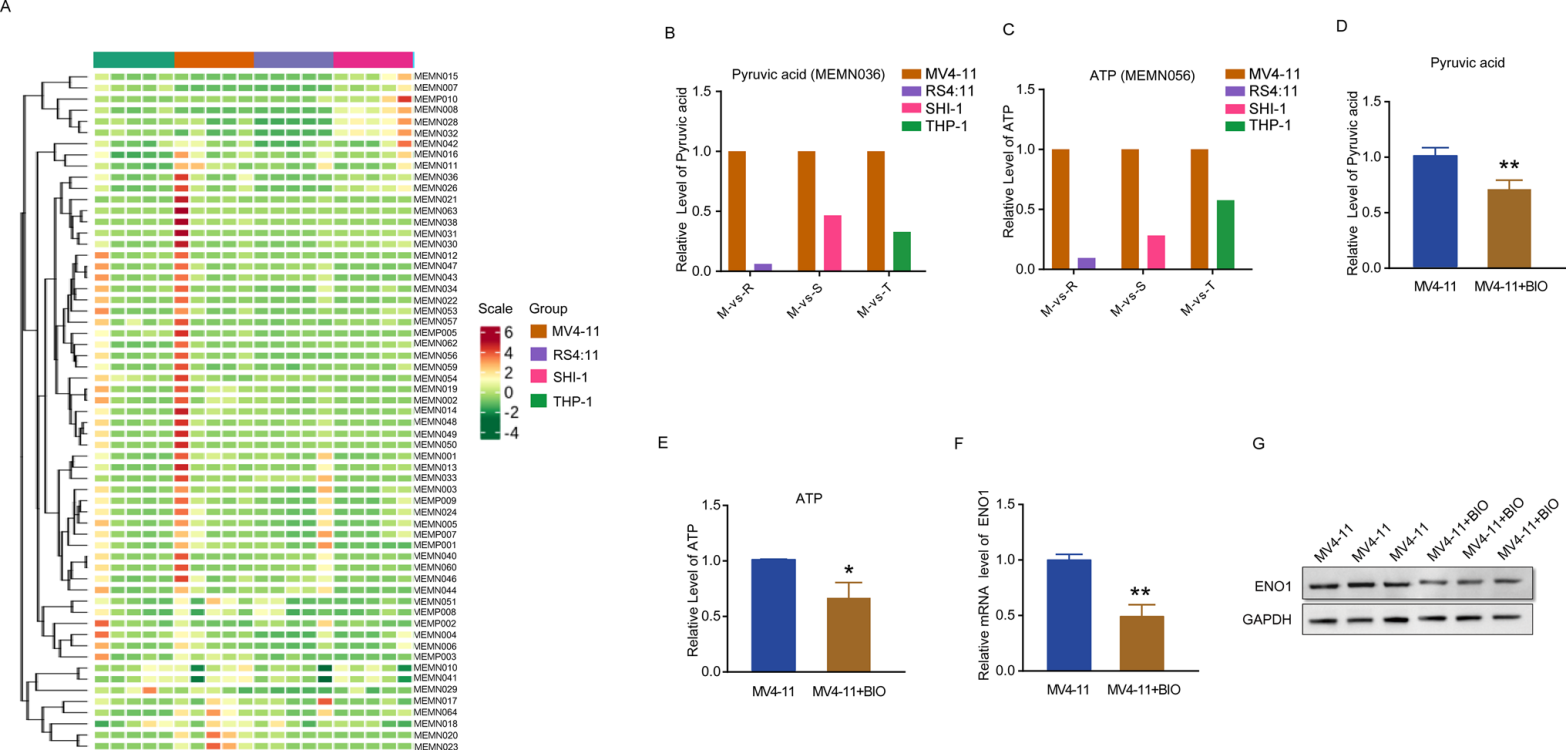
BIO represses the altered metabolite level involved in glycolysis in MV4-11 cells. **A** Heat map showing metabonomics profiling of 61 metabolites. **B** Quantification of relatively level of **B** pyruvate and **C** ATP in MV4-11 cells vs. SHI-1, THP-1 and RS4;11 cells. The **D** pyruvate and **E** ATP levels following BIO treatment were determined with the pyruvate Assay Kit and ATP Assay Kit. The **F** mRNA and **G** protein expression of ENO1 were detected using RT-qPCR and Western blot assay with or without BIO incubation. *P < 0.05, **P < 0.01

To determine the basal metabolic diversity in four leukemia cell lines including MV4-11, we performed metabolomics profiling to analyze the level of 61 metabolites involved in 3 energy metabolism pathways (Fig. 5A). The detailed results are shown in supplementary Table S1. Out of the 6 vital metabolites (D-Glucose 6-phosphate, fructose 1,6-bisphosphate, D-Fructose 6-phosphate, glycerol 3-phosphate, dihydroxyacetone phosphate and pyruvate) involved in glycolysis, pyruvate levels were elevated in MV4-11 cells compared to SHI-1, THP-1 and RS4;11 cells (Fig. 5B). Furthermore, the total ATP levels were also higher in MV4-11 cells compared with the other three cell lines (Fig. 5C), suggesting that the distinct response of MV4-11 cells to BIO administration may result from its dysregulated pyruvate and ATP metabolism. To confirm this hypothesis, we investigated the effect of BIO on pyruvate and ATP levels in MV4-11 cells using special content assay kits, and found that BIO notably reduced the pyruvate and ATP levels (Fig. 5D, E). Enolase1 (ENO1) is a glycolytic enzyme that converts 2-phosphoglycerate into phosphoenolpyruvate during aerobic glycolysis. It is well-recognized that ENO1 plays a key role in the Warburg effect and promotes proliferation, migration, and invasion of cancer cells by accelerating glycolysis. We examined the effect of BIO on ENO1 expression using RT-qPCR and Western blot and found that BIO decreased ENO1 expression at the mRNA and protein levels (Fig. 5F, G). Taken together, these findings suggest that BIO regulates apoptosis and proliferation of MV4-11 cells by inhibiting the aberrantly up-regulated glycolysis pathway.

## Discussion

In this study, we demonstrated that low concentration of GSK3β inhibitor, BIO, suppressed the proliferation of FLT3-ITD mutated cells by elevating cyclin D2 expression, reducing p21 expression, inhibiting DNA polymerase and eventually causing G1 cell cycle arrest. BIO treatment also promoted the apoptosis of representative FLT3-mutated, MV4-11 cells, through regulating the expression of caspase3 and inhibited tumor growth in vivo. Metabolomics analysis indicated that BIO regulated glycolysis, as indicated by the increased levels of pyruvate and ATP. Therefore, our findings suggest that BIO could be a potential drug for treatment of AML by regulating cancer cell growth and metastasis.

Since GSK3 is a key kinase associated with the regulation of multiple biological process including proliferation, apoptosis and differentiation of leukemia cells, its inhibitors have been considered as potential therapeutic agents for AML [22, 23]. Leukemia with FLT3-ITD mutations accounts for a rare form of AML with high rates of relapse and drug resistance. Several studies have focused on the interactive mechanisms between GSK3 and FLT3-ITD. It is initially found that Linifanib, an inhibitor of constitutive activation of FLT3, suppressed phosphorylation of GSK3β in FLT3-ITD mutant cells. Unexpectedly, inhibition of GSK3 enhanced Linifanib-induced apoptosis of ITD mutant cells, suggesting the association between GSK3 and FLT3 [24]. In contrast, Woolley et al. reported that GSK3 and FLT3 had opposing functions. In their study, PKC412, an inhibitor of FLT3, decreased p22phox, the small membrane-bound component of the Nox complex acting in the regulation of reactive oxygen species (ROS), an effect that was reversed by GSK3 inhibition [25]. Another study showed that sorafenib, a FLT3-ITD inhibitor, suppressed the activation of checkpoint kinase Chk1 to trigger DNA-damage and augmented the apoptosis of MV4-11 cells that was induced by etoposide treatment, meanwhile inactivation of GSK3 recovered durative Chk1 activation and notably abbreviated etoposide-induced apoptosis where it seems like GSK3 played opposite roles in regulating the activation of Chk1 and apoptosis of leukemia cells [26]. In addition, a genome-wide CRISPR screen revealed that reduction of GSK3 enhanced drug resistance to ACC20, a selective inhibitor of FLT3, in AML patients, suggesting that inhibition of GSK3 may reduce the efficiency of clinical treatment with FLT3 inhibitors [27]. Although the above studies investigated the potential interactive and/or synergistic association between GSK3 and FLT3, it was unclear whether GSK3 inhibitors specifically regulated the biological function of leukemia cells with FLT3-ITD and not those with other mutations.

BIO is a selective inhibitor of GSK3β that was initially demonstrated to suppress proliferation of human leukemia TF-1, HL-60, K562, and U937 cells [28]. Afterwards, experimental results showed that BIO promotes the proliferation of E26 transforming sequence-related gene (ERG)-induced K562 cells [29], indicating that GSK3 plays a role in regulating leukemia cell viability. Moreover, Wang et al. reported that BIO significantly inhibited all-trans retionic acid (ATRA)-induced apoptosis of acute promyelocytic leukemia HL60 cells [30]. Although the above studies demonstrated that BIO plays a role in regulating the growth of leukemia cells, the underlying mechanisms as well as its effects on different types of cell were unclear. Interestingly, we found that BIO selectively suppressed the proliferation of cells with FLT3-ITD mutations such as MV4-11 and MOLM13 cells, but did not affect the proliferation of leukemia cell lines harboring other types of mutations. Subsequent experiments reveled that BIO inhibited cell proliferation by arresting cells in the G1-phase. There is need to determine if BIO inhibits GSK3 function by inhibiting interaction between GSK3 and FLT3 or by affecting downstream targets of GSK3 that are associated with FLT3 function.

We then demonstrated that BIO-induced apoptosis of MV4-11 by regulating caspase3 and p53 pathways without changing other 16 intracellular proteins involved in apoptotic signaling pathways. However, these unaffected proteins were reported to be involved in the apoptosis of MV4-11 cells in other studies e.g., loss of ataxia telangiectasia mutated (ATM) mitigated the effect of miR-100 exhaustion on cell viability and apoptosis in AML cells [31], provirus integrating site moloney murine leukemia virus 3 (PIM3) overexpression enhanced AML cell proliferation and inhibited instinctive apoptosis by phosphorylating BAD (pBAD) at Ser112 [32], and Polyphyllin I affected the apoptosis of AML cell lines by regulating expression of phosphorylated-JNK [33]. Future studies are required to evaluate the precise mechanism underlying the effect of BIO on the apoptotic process and to identify downstream target proteins in the pathway triggering attenuated cell viability of AML cells with FLT3 mutation.

In addition to apoptosis, in vitro metabolomics profile analysis and metabolite detection showed that BIO also regulates cell viability by inhibiting the production of two key metabolites, pyruvate and ATP that play significant roles in the glycolysis of AML cells. Irregular metabolism of cancer cells characterized by excessive glycolysis has been shown to promote malignant proliferation and metastasis to aggravate cancer development. Increased ATP and pyruvate levels were observed in acute myeloid leukemia cells and transgenic ALL mouse model, concomitantly with high glucose uptake, glycolytic capacity and reserve, and were associated with aberrant cell proliferation resulting from uncontrolled cell cycle [34, 35]. Here, we found that the decreased levels of ATP and pyruvate following BIO treatment were partly accompanied by suppressed expression of ENO1, implying a potential strategy to restore basic energy metabolism against highly proliferative properties in AML cells with FLT3-ITD. However, further studies are required to identify the specific mechanisms underlying BIO inhibition of the inordinate glycolysis during AML development.

Collectively, we demonstrated that BIO, an effective GSK3β inhibitor, suppresses cell proliferation by arresting cells in the G1-phase in vitro and tumor growth in vivo, and induces cell apoptosis through caspase3 pathway in MV4-11 cells with FLT3 mutation. The metabolomics profiling analysis also reveals that BIO suppresses tumors by inhibiting ATP and pyruvate accumulation. These findings indicate that BIO could be an attractive treatment agent for AML with FLT3 mutations.

## Supplementary Information

Below is the link to the electronic supplementary material.

Supplementary file1 (XLSX 23 kb)

## Data Availability

The datasets generated during and/or analyzed during the current study are available from the corresponding author on reasonable request.

## References

[1] Döhner H, Weisdorf DJ, Bloomfield CD (2015). Acute myeloid leukemia. N Engl J Med.

[2] Ley TJ, Miller C, Ding L, Raphael BJ, Mungall AJ, Robertson A, Hoadley K, Triche TJ, Laird PW, Baty JD, Fulton LL, Fulton R, Heath SE, Kalicki-Veizer J, Kandoth C, Klco JM, Koboldt DC, Kanchi KL, Kulkarni S, Lamprecht TL, Larson DE, Lin L, Lu C, McLellan MD, McMichael JF, Payton J, Schmidt H, Spencer DH, Tomasson MH, Wallis JW, Wartman LD, Watson MA, Welch J, Wendl MC, Ally A, Balasundaram M, Birol I, Butterfield Y, Chiu R, Chu A, Chuah E, Chun HJ, Corbett R, Dhalla N, Guin R, He A, Hirst C, Hirst M, Holt RA, Jones S, Karsan A, Lee D, Li HI, Marra MA, Mayo M, Moore RA, Mungall K, Parker J, Pleasance E, Plettner P, Schein J, Stoll D, Swanson L, Tam A, Thiessen N, Varhol R, Wye N, Zhao Y, Gabriel S, Getz G, Sougnez C, Zou L, Leiserson MD, Vandin F, Wu HT, Applebaum F, Baylin SB, Akbani R, Broom BM, Chen K, Motter TC, Nguyen K, Weinstein JN, Zhang N, Ferguson ML, Adams C, Black A, Bowen J, Gastier-Foster J, Grossman T, Lichtenberg T, Wise L, Davidsen T, Demchok JA, Shaw KR, Sheth M, Sofia HJ, Yang L, Downing JR, Eley G (2013). Genomic and epigenomic landscapes of adult de novo acute myeloid leukemia. N Engl J Med.

[3] Rubnitz JE, Kaspers GJL (2021). How I treat pediatric acute myeloid leukemia. Blood.

[4] Reedijk AMJ, Klein K, Coebergh JWW, Kremer LC, Dinmohamed AG, de Haas V, Versluijs AB, Ossenkoppele GJ, Beverloo HB, Pieters R, Zwaan CM, Kaspers GJL, Karim-Kos HE (2019). Improved survival for children and young adolescents with acute myeloid leukemia: a Dutch study on incidence, survival and mortality. Leukemia.

[5] Arber DA, Orazi A, Hasserjian R, Thiele J, Borowitz MJ, Le Beau MM, Bloomfield CD, Cazzola M, Vardiman JW (2016). The 2016 revision to the World Health Organization classification of myeloid neoplasms and acute leukemia. Blood.

[6] Zhang Y, Yuan L (2021). Fms-like tyrosine kinase 3-internal tandem duplications epigenetically activates checkpoint kinase 1 in acute myeloid leukemia cells. Sci Rep.

[7] Drexler HG (1996). Expression of FLT3 receptor and response to FLT3 ligand by leukemic cells. Leukemia.

[8] Cao T, Jiang N, Liao H, Shuai X, Su J, Zheng Q (2019). The FLT3-ITD mutation and the expression of its downstream signaling intermediates STAT5 and Pim-1 are positively correlated with CXCR4 expression in patients with acute myeloid leukemia. Sci Rep.

[9] Wu X, Feng X, Zhao X, Ma F, Liu N, Guo H, Li C, Du H, Zhang B (2016). Prognostic significance of FLT3-ITD in pediatric acute myeloid leukemia: a meta-analysis of cohort studies. Mol Cell Biochem.

[10] Qiu QC, Wang C, Bao XB, Yang J, Shen HJ, Ding ZX, Liu H, He J, Yao H, Chen SN, Li Z, Xue SL, Liu SB (2018). The impact of FLT3 mutations on treatment response and survival in Chinese de novo AML patients. Hematology.

[11] Brandts CH, Sargin B, Rode M, Biermann C, Lindtner B, Schwäble J, Buerger H, Müller-Tidow C, Choudhary C, McMahon M, Berdel WE, Serve H (2005). Constitutive activation of Akt by Flt3 internal tandem duplications is necessary for increased survival, proliferation, and myeloid transformation. Can Res.

[12] Mizuki M, Fenski R, Halfter H, Matsumura I, Schmidt R, Müller C, Grüning W, Kratz-Albers K, Serve S, Steur C, Büchner T, Kienast J, Kanakura Y, Berdel WE, Serve H (2000). Flt3 mutations from patients with acute myeloid leukemia induce transformation of 32D cells mediated by the Ras and STAT5 pathways. Blood.

[13] Huang A, Ju HQ, Liu K, Zhan G, Liu D, Wen S, Garcia-Manero G, Huang P, Hu Y (2016). Metabolic alterations and drug sensitivity of tyrosine kinase inhibitor resistant leukemia cells with a FLT3/ITD mutation. Cancer Lett.

[14] Ju HQ, Zhan G, Huang A, Sun Y, Wen S, Yang J, Lu WH, Xu RH, Li J, Li Y, Garcia-Manero G, Huang P, Hu Y (2017). ITD mutation in FLT3 tyrosine kinase promotes Warburg effect and renders therapeutic sensitivity to glycolytic inhibition. Leukemia.

[15] Forde JE, Dale TC (2007). Glycogen synthase kinase 3: a key regulator of cellular fate. Cell Mol Life Sci.

[16] Li L, Song H, Zhong L, Yang R, Yang XQ, Jiang KL, Liu BZ (2015). Lithium chloride promotes apoptosis in human leukemia NB4 cells by inhibiting glycogen synthase kinase-3 beta. Int J Med Sci.

[17] Martelli AM, Evangelisti C, Paganelli F, Chiarini F, McCubrey JA (2021). GSK-3: a multifaceted player in acute leukemias. Leukemia.

[18] Suganuma K, Miwa H, Imai N, Shikami M, Gotou M, Goto M, Mizuno S, Takahashi M, Yamamoto H, Hiramatsu A, Wakabayashi M, Watarai M, Hanamura I, Imamura A, Mihara H, Nitta M (2010). Energy metabolism of leukemia cells: glycolysis versus oxidative phosphorylation. Leuk Lymphoma.

[19] Abrams SL, Akula SM, Meher AK, Steelman LS, Gizak A, Duda P, Rakus D, Martelli AM, Ratti S, Cocco L, Montalto G, Cervello M, Ruvolo P, Libra M, Falzone L, Candido S, McCubrey JA (2021). GSK-3β can regulate the sensitivity of MIA-PaCa-2 pancreatic and MCF-7 breast cancer cells to chemotherapeutic drugs, targeted therapeutics and nutraceuticals. Cells.

[20] Weisberg E, Boulton C, Kelly LM, Manley P, Fabbro D, Meyer T, Gilliland DG, Griffin JD (2002). Inhibition of mutant FLT3 receptors in leukemia cells by the small molecule tyrosine kinase inhibitor PKC412. Cancer Cell.

[21] Liu CC, Wang H, Wang WD, Wang L, Liu WJ, Wang JH, Geng QR, Lu Y (2018). ENO2 promotes cell proliferation, glycolysis, and glucocorticoid-resistance in acute lymphoblastic leukemia. Cell Physiol Biochem.

[22] Hu S, Ueda M, Stetson L, Ignatz-Hoover J, Moreton S, Chakrabarti A, Xia Z, Karan G, de Lima M, Agrawal MK, Wald DN (2016). A novel glycogen synthase kinase-3 inhibitor optimized for acute myeloid leukemia differentiation activity. Mole Cancer Therapeutics.

[23] Mudgapalli N, Nallasamy P, Chava H, Chava S, Pathania AS, Gunda V, Gorantla S, Pandey MK, Gupta SC, Challagundla KB (2019). The role of exosomes and MYC in therapy resistance of acute myeloid leukemia: Challenges and opportunities. Mol Aspects Med.

[24] Hernandez-Davies JE, Zape JP, Landaw EM, Tan X, Presnell A, Griffith D, Heinrich MC, Glaser KB, Sakamoto KM (2011). The multitargeted receptor tyrosine kinase inhibitor linifanib (ABT-869) induces apoptosis through an Akt and glycogen synthase kinase 3β-dependent pathway. Mol Cancer Ther.

[25] Woolley JF, Naughton R, Stanicka J, Gough DR, Bhatt L, Dickinson BC, Chang CJ, Cotter TG (2012). H2O2 production downstream of FLT3 is mediated by p22phox in the endoplasmic reticulum and is required for STAT5 signalling. PLoS ONE.

[26] Kurosu T, Nagao T, Wu N, Oshikawa G, Miura O (2013). Inhibition of the PI3K/Akt/GSK3 pathway downstream of BCR/ABL, Jak2-V617F, or FLT3-ITD downregulates DNA damage-induced Chk1 activation as well as G2/M arrest and prominently enhances induction of apoptosis. PLoS ONE.

[27] Hou P, Wu C, Wang Y, Qi R, Bhavanasi D, Zuo Z, Dos Santos C, Chen S, Chen Y, Zheng H, Wang H, Perl A, Guo D, Huang JA (2017). Genome-wide CRISPR screen identifies genes critical for resistance to FLT3 inhibitor AC220. Cancer Res.

[28] Holmes T, O'Brien TA, Knight R, Lindeman R, Shen S, Song E, Symonds G, Dolnikov A (2008). Glycogen synthase kinase-3beta inhibition preserves hematopoietic stem cell activity and inhibits leukemic cell growth. Stem cells.

[29] Mochmann LH, Bock J, Ortiz-Tánchez J, Schlee C, Bohne A, Neumann K, Hofmann WK, Thiel E, Baldus CD (2011). Genome-wide screen reveals WNT11, a non-canonical WNT gene, as a direct target of ETS transcription factor ERG. Oncogene.

[30] Wang CY, Yang TT, Chen CL, Lin WC, Lin CF (2014). Reactive oxygen species-regulated glycogen synthase kinase-3β activation contributes to all-trans retinoic acid-induced apoptosis in granulocyte-differentiated HL60 cells. Biochem Pharmacol.

[31] Sun Y, Wang H, Luo C (2020). MiR-100 regulates cell viability and apoptosis by targeting ATM in pediatric acute myeloid leukemia. Biochem Biophys Res Commun.

[32] Luo H, Sun R, Zheng Y, Huang J, Wang F, Long D, Wu Y (2020). PIM3 promotes the proliferation and migration of acute myeloid leukemia cells. OncoTargets Ther..

[33] Tian Y, Jia SX, Shi J, Gong GY, Yu JW, Niu Y, Yang CM, Ma XC, Fang MY (2019). Polyphyllin I induces apoptosis and autophagy via modulating JNK and mTOR pathways in human acute myeloid leukemia cells. Chem Biol Interact.

[34] He X, Wan J, Yang X, Zhang X, Huang D, Li X, Zou Y, Chen C, Yu Z, Xie L, Zhang Y, Liu L, Li S, Zhao Y, Shao H, Yu Y, Zheng J (2021). Bone marrow niche ATP levels determine leukemia-initiating cell activity via P2X7 in leukemic models. J Clin Investig.

[35] Chan LN, Chen Z, Braas D, Lee JW, Xiao G, Geng H, Cosgun KN, Hurtz C, Shojaee S, Cazzaniga V, Schjerven H, Ernst T, Hochhaus A, Kornblau SM, Konopleva M, Pufall MA, Cazzaniga G, Liu GJ, Milne TA, Koeffler HP, Ross TS, Sánchez-García I, Borkhardt A, Yamamoto KR, Dickins RA, Graeber TG, Müschen M (2017). Metabolic gatekeeper function of B-lymphoid transcription factors. Nature.

